# Decreased number of hospitalized children with severe acute lower respiratory infection after introduction of the pneumococcal conjugate vaccine in the Eastern Democratic Republic of the Congo

**DOI:** 10.11604/pamj.2020.37.211.22589

**Published:** 2020-11-03

**Authors:** Archippe Muhandule Birindwa, Jeanniere Tumusifu Manegabe, Aline Mindja, Rickard Nordén, Rune Andersson, Susann Skovbjerg

**Affiliations:** 1 Department of Infectious Disease, Institute of Biomedicine, University of Gothenburg, Gothenburg, Sweden,; 2 Panzi Hospital, Bukavu, Democratic Republic of Congo,; 3 Université Évangélique en Afrique, Bukavu, Democratic Republic of Congo,; 4 Department of Clinical Microbiology, Sahlgrenska University Hospital, Gothenburg, Region Västra Götaland, Sweden,; 5 Centre for Antibiotic Resistance Research (CARe), Gothenburg University, Gothenburg, Sweden

**Keywords:** Acute lower respiratory infections, case fatality, 13-valent pneumococcal conjugate vaccine, Democratic Republic of the Congo

## Abstract

**Introduction:**

acute lower respiratory infections (ALRI) are a leading killer of children under five worldwide including the Democratic Republic of the Congo (DR Congo). We aimed to determine the morbidity and case fatality rate due to ALRI before and after introduction of the 13-valent pneumococcal conjugate vaccine (PVC13) in DR Congo 2013.

**Methods:**

data were collected from medical records of children with a diagnosis of ALRI, aged from 2 to 59 months, treated at four hospitals in the Eastern DR Congo. Two study periods were defined; from 2010 to 2012 (before introduction of PCV13) and from 2014 to 2015 (after PCV13 introduction).

**Results:**

out of 21,478 children admitted to the hospitals during 2010-2015, 2,007 were treated for ALRI. The case fatality rate among these children was 4.9%. Death was significantly and independently associated with malnutrition, severe ALRI, congenital disease and symptoms of fatigue. Among the ALRI hospitalised children severe ALRI decreased from 31% per year to 18% per year after vaccine introduction (p = 0.0002) while the fatality rate remained unchanged between the two study periods. Following introduction of PCV13, 63% of the children diagnosed with ALRI were treated with ampicillin combined with gentamicin while 33% received ceftriaxone and gentamicin.

**Conclusion:**

three years after PCV13 introduction in the Eastern part of the DR Congo, we found a reduced risk of severe ALRI among children below five years. Broad-spectrum antibiotics were frequently used for the treatment of ALRI in the absence of any microbiological diagnostic support.

## Introduction

Acute lower respiratory infections (ALRI) are a leading cause of mortality and morbidity in children under five years, particularly in low- and middle-income countries [1-3]. In 2016, there were over 68 million episodes of ALRI among children younger than 5 years worldwide; this was equivalent to 0.11 cases per child-year, and more than 5 million children were hospitalised due to the infection [2]. In the Democratic Republic of the Congo (DR Congo), ALRI caused 20% of the deaths among children between 1 and 59 months in 2017 and the rate of deaths due to ALRI was 9.4 per 1,000 live births in the same age group [4]. Approximately 40% only of the children below five years with ALRI or suspected pneumonia in the DR Congo were treated by a qualified healthcare provider while the remaining children were taken care of by non-appropriate providers (e.g. private pharmacies, traditional practitioners, relatives or other services, such as shops) [5, 6].

In 2013, the 13-valent pneumococcal conjugate vaccine (PCV13) was introduced into the general vaccination program for children below one year of age in DR Congo. The vaccine was given at 6, 10 and 14 weeks of age without any catch-up campaign. In 2016, the PCV was estimated to prevent 52,000 ALRI-related deaths in children below 5 years worldwide [2]. Several published studies have documented a reduction in all manifestations of pneumococcal disease following the routine use of PCV13 or PCV10 in low- and middle-income countries [7-11]. Pneumonia, the most severe manifestation of pneumococcal-caused ALRI, is classified by the World Health Organisation (WHO) as rapid breathing and/or chest indrawings in a child between 2 and 59 months while severe pneumonia is defined as pneumonia with the addition of any general danger signs, (i.e. inability to drink, persistent vomiting, convulsions, lethargy or unconsciousness, stridor in a calm child or severe malnutrition) [12].

According to the WHO pneumonia management guidelines, children between 2 and 59 months with pneumonia should be treated with amoxicillin, while those with severe pneumonia should be given the parenteral ampicillin (or penicillin) plus the gentamicin combination for at least five days as a first-line treatment [12]. The WHO recommendations also state that children failing this first-line treatment should be offered a referral to a facility with access to appropriate second-line treatment with ceftriaxone [12]. In this study, we recorded the morbidity and fatality rates in children requiring hospital care for ALRI both before and after start of the PVC13 immunization program in DR Congo. We also assessed the antibiotic treatments used for these children. We chose to include all children with ALRI (i.e. not only pneumonia cases) because, in many cases, the patient records were not detailed enough to either exclude or confirm pneumonia according to the WHO classification. In addition, the hospital diagnoses were only based on clinical assessment by the doctors, due to limited access to X-ray and other diagnostic tools.

## Methods

### Data collection

From January 2010 to December 2015 a total of 21,552 children aged from 2 to 59 months treated at four hospitals in the South-Kivu province in the Eastern part of the DR Congo were eligible for inclusion in the study. Of these, 74 children were excluded, 47 due to missing information and 27 due to patients leaving before end of treatment, mainly because they were not able to pay for the treatment costs. Of the remaining 21,478 children data were collected for 2,007 children identified with a diagnosis of ALRI; the data was taken from medical records hand-written by physicians. For comparison between the periods both before and after introduction of PCV13 the data were divided into the following two study groups; 8,283 hospitalized children during 2010-2012 (i.e. before introduction of PCV13) and 9,713 during 2014-2015 (i.e. after PCV13 introduction). During 2013 (the year of PCV13 vaccine introduction) data from 3,482 cases were not included in the comparison between the pre- and post-vaccine period. However, these were included in the overall description of ALRI, case fatalities and antibiotic treatment assessments.

### Study areas

The study was performed at two general referral hospitals, Panzi Hospital and Ciriri Hospital and two district hospitals, Miti Murhesa and Nyantede. Panzi Hospital, located in Bukavu town, is a teaching hospital that served a population of 453,000 inhabitants including 86,000 (19%) children below 5 years at the time of the study. The general referral hospital, Ciriri, located in the suburban area of Bukavu, served a population of 337,000 inhabitants including 71,000 (21%) children below five years. The other two district hospitals were located in rural areas; the Miti Murhesa district hospital served a population of 231,000 inhabitants including 52,000 (23%) children below 5 years, and the Nyantende Hospital, 132,000 inhabitants including 29,000 (22%) children [6].

### Definitions

Cases were considered as ALRI if the medical records contained any of the following discharge diagnoses: pneumonia, atypical pneumonia, bronchopneumonia, bronchitis, bronchiolitis, rhino-bronchitis, rhino-bronchiolitis, rhino-pharyngo-bronchitis, bronchopneumonia or any combination of these, with or without association with upper respiratory tract infection as assessed by the physician at the admission or at discharge from the hospital. A case was considered as severe ALRI if the medical records had a discharge diagnosis of severe or very severe pneumonia, or pneumonia with the addition of any general danger signs (not able to drink, persistent vomiting, convulsions, lethargy or unconsciousness, stridor in a calm child or severe malnutrition). The International Statistical Classification of Diseases and Related Health Problems was not used in the DR Congo and was therefore not applicable. Data were collected on age, sex, admission dates, duration of hospital care and outcome (i.e. death, complications, improvement, and cure). Recorded symptoms included the following: rapid breathing (defined as >60 respiratory rates (RR) per minute in children below 3 months, > 50 RR/min in children 3-12 months and > 40 RR/min in children >12-59 months), breathing difficulty, fever, cough or recent history of cough, weakness or fatigue, diarrhoea, vomiting, chest indrawings. Results from lung auscultations were recorded; the presence of crackles and/or rhonchi were considered as abnormal auscultation. The data were also collected on underlying conditions including malnutrition, HIV, sickle cell disease, cerebral palsy, post neonatal anoxia or congenital diseases and also the use of antibiotics and oxygen. X-ray was not used for confirming the diagnosis of pneumonia as only two of the selected hospitals (Panzi and Ciriri) had the required facilities.

### Statistical analysis

Descriptive analysis was performed by SPSS package (version 23.0) for logistic regression to analyse relationship between death and socio-demographic factors (age, sex, year of hospitalisation), duration of hospital stay, antibiotic treatments and underlying conditions. Prevalence rates with a 95% confidence interval (CI) were calculated to compare the morbidity and case fatality rates both before and after introduction of PCV13. Associations between categorical variables were analysed using the Pearson Chi-square or Fisher´s exact test, where appropriate. A p-value < 0.05 was considered statistically significant. Potential variables associated with the impact of PCV13 on pneumococcal pneumonia were assessed by odd ratios (OR) with a 95% CI.

### Ethics approval and consent to participate

The study was approved by the Commission Institutionelle d´Ethique (CIE) of the Université Catholique de Bukavu (N/Ref: (UCB/CIE/NC/06/2015) in accordance with existing ethical guidelines in DR Congo. The four Directors of the included hospitals in the study were informed and approved the study.

### Availability of data and material

The datasets used and analysed during the current study are available from the corresponding author upon reasonable request.

### Funding

This study was supported by funds at the Sahlgrenska Academy, University of Gothenburg. The funding body had no role in the design of the study, the collection, analysis or interpretation of data, nor in writing of the manuscript.

## Results

### Characteristics of the study population

**Figure 1:**
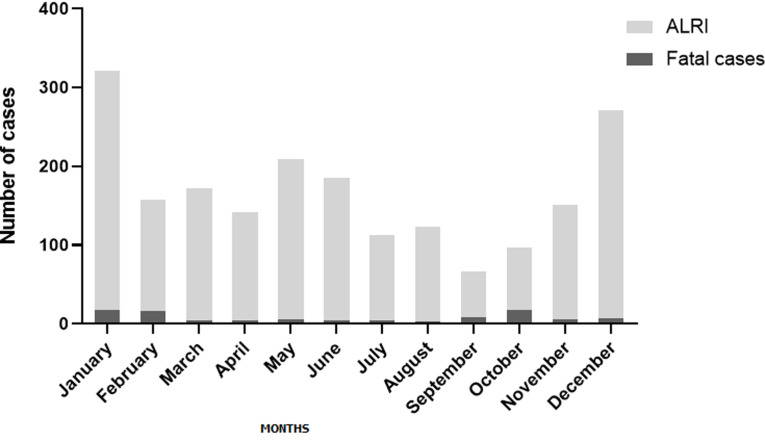
frequencies of acute lower respiratory infections (ALRIs) and fatal outcome by month in children 2-59 months of age treated due to ALRI at four different hospitals in the east of DR Congo between 2010 and 2015

A total of 2,007 (9.4%) children aged from 2 to 59 months (median of 11 months) were treated for ALRI out of 21,478 children hospitalised in the paediatric wards during 2010-2015 at four different hospitals in the south-Kivu province of Eastern DR Congo. Of these ALRI cases, 542 (27.0%) children had severe ALRI, while the remaining 1,465 (73.0%) had a diagnosis defined as non-severe ALRI (Table 1). Clinical characteristics and socio-demographic data for all children with ALRI are presented in Table 1 and Table 2. Hospitalisation due to ALRI peaked in January (n = 321, 16.0%) and December (n = 271, 13.5%) (Figure 1). The ALRI population mortality rate was estimated at 0.08 children per 100,000 inhabitants per year. The overall fatality rate in the hospitalised children with ALRI was 4.9%, and the highest fatality rate (17.5%) occurred in October (Figure 1). All but one child presented with symptoms of cough or had a history of acute cough, abnormal auscultation upon physical examination and fever (Table 1). Fatigue or weakness was found in 74.1% of ALRI cases and rapid breathing or breathing difficulty in 43.2% (Table 1). Pleural effusion was the most common complication affecting 1.0% of cases. Half of the cases (50.4%) were hospitalised for three to seven days (Table 1).

**Table 1:** socio-demographic data and clinical characteristics of the children treated at the hospitals due to acute lower respiratory infection during 2010-2015

		Number of children (%) (N=2,007)
Hospital	**CIRIRI**	472 (23.5)
	**NYANTENDE**	682 (34.0)
	**MITIMURHESA**	590 (29.4)
	**PANZI**	263 (13.1)
	**Total**	2,007 (9.4)
Sex	**Girl**	960 (47.8)
	**Boy**	1,047 (52.2)
Age (months)	**2-6**	508 (25.3)
	**7-12**	622 (31.0)
	**13-24**	481 (24.0)
	**25-36**	190 (9.5)
	**37-59**	206 (10.3)
Symptoms	**Cough or history of coughing**	2,006 (100)
	**Abnormal auscultation**	2,007 (100)
	**Fever or history of fever**	2,007 (100)
	**Rapid or difficult breathing**	868 (43.2)
	**Weakness or fatigue**	1,487 (74.1)
	**Diarrhoea/vomiting**	117 (5.8)
	**Other symptoms a**	86 (4.3)
Nutritional Status	**Malnutrition b**	238 (11.9)
Congenital diseases c		30 (1.5)
HIV		25 (1.3)
ALRI	**Non-severe ALRI**	1,465 (73.0)
	**Severe ALRI**	542 (27.0)
Antibiotic used	**Ampicillin and gentamicin**	1,313 (65.4)
	**Ceftriaxone and gentamicin**	598 (29.8)
	**Ciprofloxacin**	70 (3.5)
	**Chloramphenicol**	26 (1.3)
Oxygen used	**Nasal oxygen**	228 (11.4)
Duration of hospital stay (days)	**0-2**	107 (5.3)
	**3-7**	1,012 (50.4)
	**>8**	888 (44.2)
Outcome	**Pleural effusion as complication**	21 (1.0)
	**Improved and cured**	1,908 (95.1)
	**Death**	99 (4.9)
	**Death before 48 hours**	66 (3.3%)
	**Death after 48 hours**	33 (1.7%)

a) Other symptoms: cutaneous-mucous pallor, jaundice, skin rash, seizures, loss of consciousness. b) Malnutrition: A Z-score cut-off point of <-2 SD to classify low weight-for-age. c) Congenital diseases: sickle cell diseases, congenital heart diseases, clinical Downs syndrome, clinical malformation of legs or arms. ALRI = acute lower respiratory infection

**Table 2:** socio-demographic characteristics of hospitalized children with ALRI during 2010-2015

		Cases with ALRI/N total inpatients (%) (N=21,478)
Hospital	**CIRIRI**	472/2,617 (18.0)
	**NYANTENDE**	682/5,981 (11.4)
	**MITIMURHESA**	590/7,387 (7.9)
	**PANZI**	263/5,493 (4.8)
	**Total**	2,007/21,478 (9.4)
Sex	**Girls**	960/9,693 (9.9)
	**Boys**	1,047/11,785 (8.9)
Age (months)	**2-6**	508/ 4,701 (10.8)
	**7-12**	622/4,519 (13.8)
	**13-24**	481/3,946 (12.2)
	**25-36**	190/2,539 (7.5)
	**37-59**	206/2,291 (9.0)

### Morbidity and case fatality rate due to ALRI both before and after start of PVC13

Out of the 8,283 children between 2 and 59 months treated at any of the hospitals during the first study period (2010-2012), 1,009 cases (12.2% of all admissions) were treated for ALRI while 693/9,713 cases (7.1% of all admissions) were treated for ALRI during the period after the introduction of PCV13 (2014-2015) (*p*< 0.0001). Severe ALRI for hospitalised children decreased from 103/336 (31%) per year to 64/347 (18%) per year after vaccine introduction (*p*= 0.0002) while the total average number of ALRI cases remained unchanged between the two periods (Table 3). In children below 24 months of age the proportion of all ALRI cases decreased after PCV13 introduction, but this remained unchanged in the older children (Table 3). The proportion of cases with rapid breathing or breathing difficulty also decreased after introduction of the vaccination from 45.8%to 35.4%; *p*= 0.016) while the case fatality rate remained similar between the two study periods (Table 2).

**Table 3:** morbidity and case fatalities of acute lower respiratory infections (ALRI) by year mean (annual average) both before (2010-2012) and after (2014-2015) start of PVC13 immunization

		Before PCV13 Annual average	After PCV13 Annual average		
		ALRI cases/N inpatients (%)	ALRI cases/N inpatients (%)	OR (CI=95%)	p-value
Hospital	**CIRIRI**	86/394 (21.8)	75/556 (13.5)	0.61 (0.44-0.86)	0.0049
	**NYANTENDE**	125/926 (13.5)	104/1,202 (8.7)	0.64 (0.48-0.84)	0.0015
	**MITIMURHESA**	83/694 (12.0)	123/2,033 (6.0)	0.50 (0.37-0.67)	<0.0001
	**PANZI**	43/747 (5.8)	46/1,114 (4.1)	0.71 (0.47-1.09)	0.126
	**TOTAL**	336/2,761 (12.2)	347/4,857 (7.1)	0.58 (0.50-0.68)	<0.0001
Sex	**Girls**	150/1,344 (11.2)	172/2,363 (7.3)	0.65 (0.51-0.82)	0.0003
	**Boys**	187/1,417 (13.2)	175/2,494 (7.0)	0.53 (0.42-0.66)	<0.0001
Age (months)	**2-6**	88/675 (13.0)	78/1,338 (5.8)	0.44 (0.32-0.61)	<0.0001
	**7-12**	113/659 (17.1)	99/1,272 (7.8)	0.45 (0.34-0.60)	<0.0001
	**13-24**	75/612 (12.3)	92/1,056 (8.7)	0.71 (0.51-0.97)	0.037
	**25-36**	31/472 (6.6)	34/562 (6.0)	0.92 (0.55-1.52)	0.74
	**37-59**	29/344 (8.4)	45/630 (7.1)	0.84 (0.52-1.37)	0.50
		ALRI cases (%) (N=336)	ALRI cases (%) (N=347)		
Symptoms	**Cough or history of coughing**	336 (100)	347 (100)	1.03(0.02-52.19)	0.98
	**Abnormal auscultation**	336 (100)	347 (100)	1.03(0.02-52.19)	0.98
	**Fever or history of fever**	336 (100)	347 (100)	1.03(0.02-52.19)	0.98
	**Rapid or difficult breathing**	154 (45.8)	123 (35.4)	0.68 (0.50-0.93)	0.016
	**Weakness or fatigue**	250 (74.4)	250 (72.0)	0.88 (0.63-1.24)	0.48
	**Diarrhoea or vomiting**	19 (5.7)	22 (6.3)	1.12 (0.59-2.12)	0.70
	**Other symptoms a**	18 (5.4)	10 (2.9)	0.52 (0.23-1.15)	0.10
Nutritional Status	**Malnutrition b**	35 (10.4)	48 (13.8)	1.38 (0.86-2.19)	0.17
Congenital diseases c		3 (0.9)	6 (1.7)	1.95 (0.48-7.87)	0.34
HIV		3 (0.9)	5 (1.4)	1.62 (0.38-6.82)	0.50
ALRI	**Non-severe ALRI**	234 (69.6)	283 (81.6)	1.92 (1.34-2.75)	0.0003
	**Severe ALRI**	103 (30.7)	64 (18.4)	0.51 (0.35-0.73)	0.0002
Antibiotic used	**Ampicillin and gentamicin**	232 (69.0)	219 (63.1)	0.76 (0.55-1.05)	0.10
	**Ceftriaxone and gentamicin**	88 (26.2)	114 (32.9)	1.46 (1.04-2.03)	0.025
	**Ciprofloxacin**	11 (3.3)	12 (3.5)	1.05 (0.46-2.43)	0.89
	**Chloramphenicol**	5 (1.5)	3 (0.9)	0.55 (0.13-2.43)	0.45
Oxygen used	**Nasal oxygen**	38 (11.3)	39 (11.2)	1.08 (0.67-1.76)	0.72
Duration of hospital stay (days)	**0-2 days**	16 (4.8)	21 (6.0)	1.28 (0.66-2.51)	0.45
	**3-7 days**	174 (51.8)	183 (52.7)	1.03 (0.76-1.40)	0.80
	**>8 days**	146 (43.5)	144 (41.5)	0.92 (0.68-1.25)	0.60
Outcome	**Death**	16 (4.8)	17 (4.9)	1.06 (0.52-2.13)	0.86
	**Pleural effusion**	6 (1.8)	1 (0.3)	0.15 (0.02-1.32)	0.08
	**Improved and cured**	320 (95.2)	330 (95.1)	0.97 (0.48-1.95)	0.94

### Fatality risk factors

Out of the 2,007 children treated for ALRI during the whole 6-year period from 2010 to 2015, 99 (4.9%) died due to the disease. The case fatality rate was significantly associated with severe ALRI (OR 28.58; 95% CI 14.74-55.42; p < 0.0001) (Table 4). Children hospitalised for ALRI and also had malnutrition or a congenital disease as underlying conditions, had a 9.8 and 12.4 times greater risk of dying, respectively (Table 4). The case fatality rate was 3.9 times higher in children with symptoms of fatigue (Table 4).

**Table 4:** identified fatality risk factors among hospitalised children with acute lower respiratory infections (ALRI) (n=2,007) during the period between 2010-2015

			Univariable analysis		Multivariable Analysis	
Risk factors		Death	Unadjusted OR (CI=95%)	p-value	Adjusted OR (CI = 95%)	p-value
Age (months)	**37-59**	7/199	1			
	**25-36**	7/183	1.08 (0.37-3.16)	0.87	-	-
	**13-24**	18/463	1.10 (0.45-2.69)	0.82	-	-
	**7-12**	28/594	1.36 (0.58-3.16)	0.47	-	-
	**2-6**	39/469	2.36 (1.03-5.37)	0.04	-	-
					
Sex	**Girls**	47/913	1			
	**Boys**	52/995	1.01 (0.67-1.52)	0.94	-	-
					
Severe ALRI	**No**	10/1455	1			
	**Yes**	89/453	28.58 (14.74-55.42)	<0.0001	139.06 (86.94-145.8)	<0.0001
					
Malnutrition a	**No**	48/1721	1			
	**Yes**	51/187	9.77 (6.41-14.91)	<0.0001	8.03 (3.18-20.28)	<0.0001
					
Congenital diseases b	**No**	88/1889	1			
	**Yes**	11/19	12.42 (5.73-26.91)	<0.0001	9.85 (1.76-55.28)	<0.0001
					
Rapid breathing c	**No**	28/1083	1			
	**Yes**	71/740	3.71 (2.37-5.80)	<0.0001	1.38 (0.38-4.94)	0.61
					
Fatigue	**No**	9/487	1			
	**Yes**	90/1237	3.93 (1.96-7.87)	0.0001	2.75 (0.63-11.92)	0.01

### Antibiotic treatments of pneumonia

Among the children with non-severe ALRI, 1175/1465 (80.2%) patients were treated with the ampicillin and gentamicin combination, (i.e. the treatment recommended by the WHO for severe pneumonia, while the second-line treatment ceftriaxone combined with gentamicin was used for 254/1465 (17.3%) patients. Among the children with severe ALRI, 344/542 (63.5%) were treated with the ceftriaxone and gentamicin combination while 138/542 (25.5%) were treated with the ampicillin and gentamicin combination. After the introduction of PCV13 vaccine, the use of ceftriaxone combined with gentamicin increased significantly whereas there was a slight (non-significant) decrease for the ampicillin and gentamicin combination (Table 3). During the 6-year study period, there was considerable use of antibiotics not recommended for pneumonia. This included 70 (3.5%) patients treated with ciprofloxacin, 18 of whom were non-severe ALRI cases. In addition, 26 (1.3%) were treated with chloramphenicol, 18 of whom were children with non-severe ALRI (Table 1).

## Discussion

The proportion of children aged 2-59 months admitted to hospitals in the Eastern part of the DR Congo with acute lower respiratory infections (ALRI) was 9.4% out of a total of 21,478 all-cause hospitalisations during the study period 2010-2015. This proportion is lower compared to other low-income countries, and could be due to less accessibility to hospital care in the Eastern part of the DR Congo [13, 14]. Differences between studies may also depend on variations in the definitions of ALRI. In this study, the ALRI diagnoses were defined by more than ten different physicians from various different medical backgrounds with limited access to diagnostic tools, including X-ray and microbiological diagnostic tools. Due to the retrospective study design we did not consider the WHO classification of pneumonia to be applicable, rather we chose to use the broader terms, ALRI and severe ALRI instead of pneumonia and severe pneumonia, respectively.

Most cases accrued in December and January, while most ALRI-related deaths occurred in October. The rainy season starts in September and ends in June leading to heavy rainfalls in the region, often accompanied by viral ALRI outbreaks [15-17]. Viral infections can cause severe acute respiratory infections in children. They may also be associated with secondary bacterial infections, in particular with pneumococcal bacteria [18]. We found an ALRI-related hospital mortality rate of 0.08 children per 1,000 live births while in 2016, the overall child mortality rate for DR Congo was 94.3 deaths per 1,000 live births in children below five years of age [19]; the figures were as high as 125 deaths per 1,000 live births in the South-Kivu province of DR Congo in 2014 [6]. This indicates a low mortality rate in children treated in hospitals but that only few of the sick children actually reach the hospitals. Up to 80% of deaths in severe respiratory infections may occur outside hospitals [14], indicating the need for a qualified primary healthcare system, in remote as well as urban areas.

The case fatality rate of 4.9% in our study was lower compared to studies from Malawi and Nigeria, both showing rates of about 10% [20, 21], and was much lower than the rates reported for hospitalised children in Mauritania (18%) [22]. Conversely, lower case fatality rates were reported in South Africa (2%) [23]. In the present study, hospitalisation due to severe ALRI conferred 28 times higher risk for fatal outcome compared to non-severe ALRI. Similar results were found in Malawi, Nigeria and in Bangladesh, where risk of death was greatest in children between 2 and 11 months and those with very severe pneumonia [20, 21, 24]. This might be explained by the quality of healthcare standards and organisation, the accessibility and the absence of clinical guidelines [25].

We further identified malnutrition and congenital disease as underlying conditions increasing the risk of fatal outcome in ALRI. Malnutrition is an important co-factor for severe infections; it is associated with immune-deficiency and respiratory muscle atrophy and can triple the mortality risk of pneumonia [26, 27]. Malnutrition has previously been associated with death in childhood pneumonia in several countries, including the Central African Republic, India, Brazil and Bangladesh [24, 28-30]. Diminished immune function via reduced production and/or diminished function of the immune system cellular components is an explanation for the increased incidence and severity of infections in malnourished children [31]. We further found the symptom of fatigue to be an independent risk factor for death. Weakness or fatigue can be found in most severely ill children; however, the evaluation of this symptom can differ from one physician to another, especially if no standardized definition exists.

Our results support the evidence that PCV13 reduces the frequency of hospital care due to severe ALRI [8, 32-34]. The proportion of severe ALRI cases decreased from 31% on average per year to 18% on average per year after introduction of PCV13. In addition, the proportion of all ALRI cases decreased in children below 24 months of age but remained unchanged in older children. Most of the children older than 3 years in the region had not received PCV13 at the time of the study. PCV13 impact studies in countries supported by the Vaccine Alliance GAVI (Burkina Faso, Gambia, Lao PDR, Mongolia, Malawi, Papua New Guinea, Rwanda and Togo) have demonstrated that, two years after PCV13 introduction, pneumonia hospitalisations were significantly reduced by 33% in children below 12 months of age and by 26% in children between 12 and 23 months of age [7, 35]. At the Children´s Hospital in Montevideo, Uruguay, the hospitalisation rates for community-acquired pneumonia and pneumococcal pneumonia decreased significantly in all children by 56% and 43%, respectively, both between the pre-vaccine years (2005-2007) and during the year after vaccination introduction (2009) [36]. Furthermore, in Malawi, a considerable decrease in severe pneumonia cases and death was shown in children after PCV13 introduction [37]. In this study, the relatively low case fatality rate in children admitted to hospital for ALRI remained unchanged both before and after PCV13 introduction. Since many critically-ill Congolese children never reach the hospitals a community-based study could hypothetically have arrived at a different conclusion on the impact of PCV13.

We found that the use of ceftriaxone in combination with gentamicin for ALRI was high; also, there was a tendency towards increased use after introduction of PCV13 vaccine while there was a tendency towards decreased use of ampicillin in combination with gentamicin. The WHO guidelines recommend the use of the ceftriaxone and gentamicin combination for pneumonia cases showing no improvement on treatment with the ampicillin and gentamicin combination [12]. It was not possible to evaluate the rationale for antibiotic use since no bacteriologic cultures were performed in our study cohort. Nevertheless, our data revealed the use of ciprofloxacin for 70 (3.8%) patients and chloramphenicol for 26 (1.4%) patients despite this not being recommended for the treatment of pneumonia. Moreover, we have recently shown a high level of resistance to commonly-used antibiotics in the region (i.e. penicillin, co-trimoxazole and ceftriaxone) for pneumococci carried in the nasopharynx of healthy children [38]. This highlights the need for a more rational use of antibiotics in the region.

## Conclusion

Three years after PCV13 introduction in the Eastern part of the DR Congo, we found a decrease in severe ALRI in children below five years. Children with severe ALRI, or those with underlying conditions including malnutrition or congenital disease had a higher risk of death compared to those hospitalised for non-severe ALRI or those without any underlying conditions. We highlight the inappropriate use of antibiotics, such as chloramphenicol and ciprofloxacin for ALRI especially in the absence of any diagnostic microbiological support.

### What is known about this topic

- PCV13 have been shown to reduce severe childhood respiratory infections in other parts of Sub-Saharan Africa;
- Ampicillin combined with gentamicin is the recommended first line treatment of severe pneumonia.

### What this study adds

- We found a significant reduction of children hospitalized due to severe acute lower respiratory infections (ALRI) after introduction of PCV13 in the infant vaccination program in the Eastern part of DR Congo;
- That risk factors such as malnutrition, congenital diseases and severe ALRI were associated with case fatality in hospitalized children with ALRI in the region;
- There is an inappropriate use of antibiotics for ALRI, such as chloramphenicol and ciprofloxacin, especially in the absence of any microbiological diagnostic support.
